# Effect of home exercise on prevention and treatment of lymphedema in breast cancer patients

**DOI:** 10.3389/fonc.2025.1665012

**Published:** 2025-12-16

**Authors:** Hui Yin, Weiwei Wu, Qungui Zhang, Fangfang Xie

**Affiliations:** 1 Medical Imaging Department, Ganzhou Cancer Hospital, Ganzhou, Jiangxi, China; 2 Department of Oncology, Ganzhou Cancer Hospital, Ganzhou, Jiangxi, China; 3 Rheumatology and Immunology, Ganzhou People’s Hospital, Ganzhou, Jiangxi, China

**Keywords:** breast cancer, home exercise, lymphedema, quality of life, upper limb function

## Abstract

**Objective:**

To develop a systematic home exercise intervention program based on the Expert Consensus on Home Exercise for Prevention and Treatment of Lymphedema in Postoperative Breast Cancer Patients and evaluate its effectiveness in preventing and treating lymphedema in breast cancer patients, thereby improving their home-based quality of life.

**Methods:**

A pre–post controlled study was conducted involving 104 breast cancer patients who underwent surgery at Ganzhou Cancer Hospital between November 2024 and May 2025. Participants were chronologically assigned to a control group (n = 52, receiving routine care) or an experimental group (n = 52, receiving routine care plus a consensus-based systematic home exercise intervention). The intervention, delivered by a multidisciplinary team, included assessment of exercise contraindications, evaluation of lymphedema status, exercise capacity testing, and implementation of resistance training, flexibility exercises, aerobic exercise, deep breathing exercises, and self-manual lymphatic drainage.

**Results:**

The incidence of lymphedema was significantly lower in the experimental group (7.69%) than in the control group (34.62%), with an absolute risk reduction of 26.92% (95% CI: 12.51% to 41.33%). The experimental group also demonstrated significantly greater improvement in the Disabilities of the Arm, Shoulder, and Hand (DASH) score (mean difference = −12.90 points; 95% CI: −15.80 to −10.00) and the Functional Assessment of Cancer Therapy-Breast (FACT-B) score (mean difference = +13.30 points; 95% CI: 10.49 to 16.11). Exercise compliance was significantly higher in the experimental group (96.15% *vs*. 78.85%).

**Conclusion:**

The consensus-based systematic home exercise intervention effectively reduced lymphedema incidence, improved upper limb function, and enhanced quality of life in breast cancer patients. The program demonstrates high clinical feasibility and is recommended for wider application. Future multi-center randomized controlled trials are warranted to further validate its long-term benefits.

## 1 Introduction

Breast cancer-related lymphedema (BCRL) is a prevalent chronic complication following breast cancer surgery, arising from lymphatic system dysfunction caused by surgery, radiotherapy, or tumor metastasis, leading to the accumulation of protein-rich lymph fluid in the interstitial spaces (1). Lymphedema causes limb dysfunction and pain, significantly increases patients’ psychological burden, and diminishes their quality of life (2). While exercise has been shown to promote functional recovery of the affected limb and improve emotional well-being (3), clinical awareness of home exercise management among both healthcare providers and patients remains insufficient. Limited medical resources further highlight the urgent need for a scientifically grounded and clinically feasible management protocol.

The Expert Consensus on Home Exercise for Prevention and Treatment of Lymphedema in Postoperative Breast Cancer Patients (hereafter referred to as the “Consensus”) provides an evidence-based foundation for home exercise management (4). Building upon this Consensus and integrating clinical experience, this study aimed to evaluate the effectiveness of a systematic home exercise intervention for the prevention and treatment of lymphedema in breast cancer patients, thereby validating the clinical utility of the Consensus and providing evidence to support standardized home exercise management.

## 2 Materials and methods

### 2.1 Study participants

**Figure 1:**
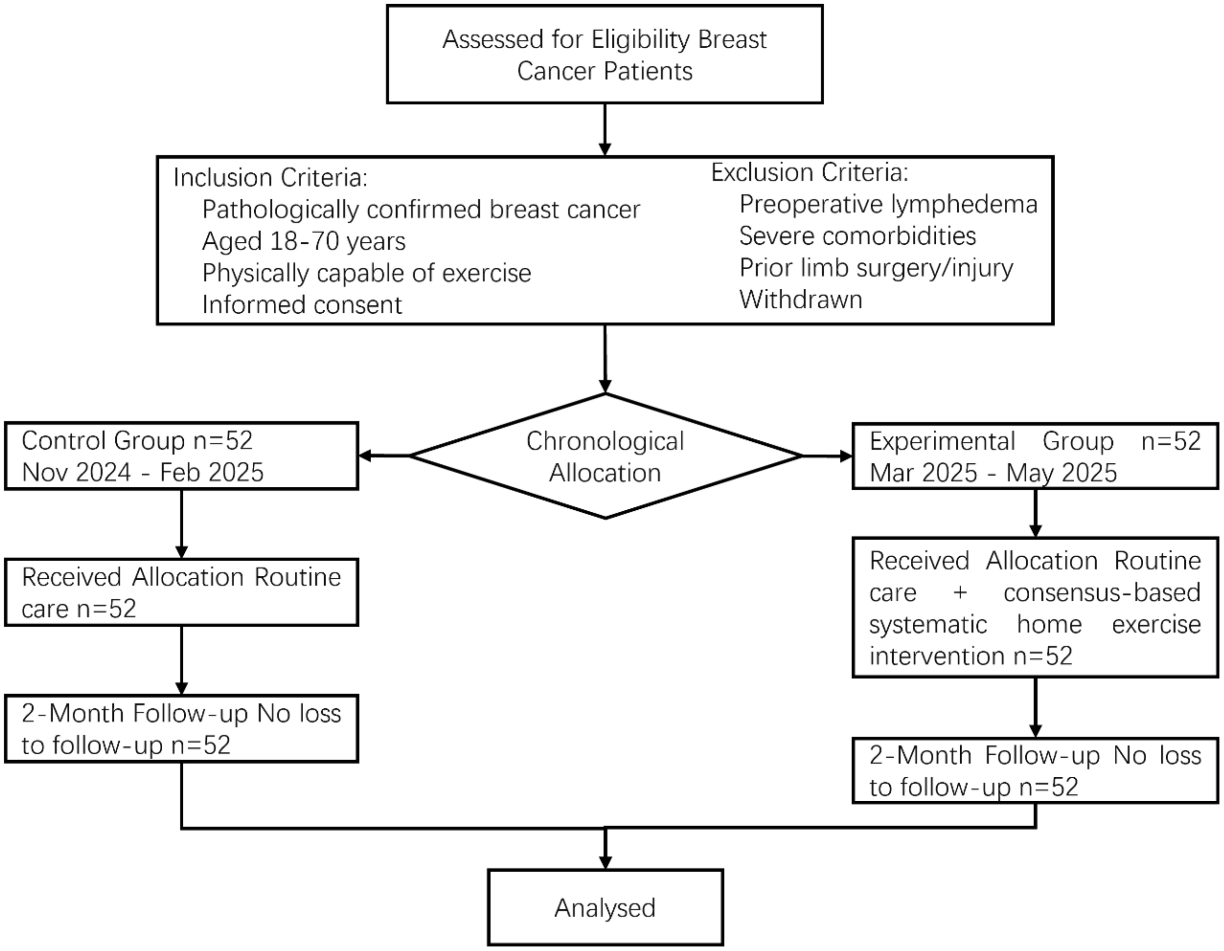
CONSORT flow diagram of participant progression through the study.

A pre–post controlled study design was employed. Participants were breast cancer patients who underwent surgery at Ganzhou Cancer Hospital between November 2024 and May 2025. Chronological assignment was used: the control group included patients operated on between November 2024 and February 2025 (n = 52), and the experimental group included those operated on between March 2025 and May 2025 (n = 52). The inclusion criteria were as follows: 1) pathologically confirmed breast cancer patients who underwent surgical treatment (e.g., modified radical mastectomy and axillary lymph node dissection); 2) aged 18–70 years, cognitively intact, and physically capable of exercise; 3) well-healed postoperative incisions without serious complications; and 4) provided voluntary informed consent to participate. The exclusion criteria were as follows: 1) preoperative diagnosis of lymphedema or presence of edema due to cardiac, renal, or nutritional causes; 2) severe cardiovascular or cerebrovascular diseases, arthritis, or other comorbidities; 3) history of prior surgery or injury to the affected limb; and 4) withdrawal from the study. The flow of participants through each stage of the study is presented in the Consolidated Standards of Reporting Trials (CONSORT) diagram (**Figure 1**). Baseline characteristics were comparable between the two groups (p > 0.05), as shown in **Table 1**.

**Table 1:** Baseline characteristics of the study groups.

Item	Control group (n = 52)	Experimental group (n = 52)	Statistic (t/χ^2^/Z^*^)	P-value
Age (years, mean ± SD)	42.3 ± 15.5	41.9 ± 12.9	1.395	0.279
Educational level, n (%)			−0.027	0.916
Junior high school and below	11	10		
High school and junior college	29	32		
Bachelor's degree and above	12	10		
Pathological type, n (%)			0.087	0.788
Invasive	43	40		
Non-invasive	9	12		
Clinical stage, n (%)			−0.662	0.523
Stage I	10	11		
Stage II	28	30		
Stage III	12	8		
Stage IV	2	3		

^*^Z-values from Mann–Whitney U test.

### 2.2 Study methods

#### 2.2.1 Control group

Patients received standard postoperative care, including wound management, discharge instructions (e.g., avoiding heavy lifting on the affected side and infection prevention), and basic lymphedema prevention measures (e.g., recognizing early symptoms, limb protection and skin care, appropriate functional exercises, and healthy lifestyle guidance). They did not receive the structured home exercise intervention.

#### 2.2.2 Experimental group

In addition to routine care, patients received the systematic home exercise intervention based on the Consensus (4), implemented as follows. Assessment phase—exercise contraindication screening: Patients were screened for conditions precluding exercise participation (e.g., extreme fatigue, severe anemia, ataxia, disease progression, infection, unhealed wounds, and bone metastasis). Lymphedema status evaluation: Patients were assessed for edema stability. Exercise was not recommended during the unstable phase (defined as receiving edema treatment within the past 3 months, active arm infection requiring antibiotics, recent decline in daily activity, or limb circumference change ≥10%). Exercise capacity testing: Pre-exercise assessments included cardiopulmonary endurance (e.g., 6-minute walk test), muscle strength (e.g., grip strength), flexibility, and balance [e.g., Short Physical Performance Battery (SPPB), timed up-and-go test, sit-to-stand test, and daily walking speed].

A multidisciplinary team (including surgeons, rehabilitation physicians, exercise therapists, lymphedema therapists, physiotherapists, and oncology nurses) monitored the patients’ home exercise plans. Patients learned the structured exercise program using manuals and video tutorials to ensure safe and effective implementation. To ensure implementation fidelity, the multidisciplinary team conducted periodic checks via WeChat video calls to visually assess exercise form and self-manual lymphatic drainage (self-MLD) technique. Patients were also encouraged to submit video recordings of their exercises for qualitative review and feedback. High-risk patients wore compression sleeves during exercise (5).

Home exercise program: 1) Resistance training: utilized bodyweight or tools (resistance bands and dumbbells). Intensity: 50%–80% of one-repetition maximum (1RM). Volume: 8–12 repetitions per exercise, two to three sets, 1-minute rest between sets. Frequency: 2–3 times/week. 2) Flexibility training: included yoga, Pilates, Qigong, and stretching exercises, often combined with breathing techniques. Duration: 30–60 minutes/session, adjusted based on patient tolerance. 3) Aerobic exercise: included brisk walking, jogging, cycling, hiking, Tai Chi, and stair climbing. Intensity: moderate. Duration: 30–60 minutes/day or 150–300 minutes/week. 4) Deep breathing exercises: integrated with other exercises. Technique: deep inhalation during muscle contraction and exhalation during relaxation to modulate intrathoracic pressure and enhance lymphatic return. 5) Self-MLD: techniques taught by trained lymphedema therapists. Performed twice daily (morning and evening), 15–20 minutes/session, or once post-exercise; affected limb elevated appropriately. Patients could combine different exercise modalities (e.g., resistance + aerobic) to comprehensively promote lymphatic return, improve function, and accommodate preferences and endurance (6).

### 2.3 Outcome measures

Outcomes were assessed 1 month post-intervention by two trained researchers via face-to-face evaluation. 1) Lymphedema incidence: measured using a non-elastic tape (accuracy 0.1 cm). Patient stood with arms relaxed; circumference was measured at fixed points (10 cm above and below the elbow crease). Measurements were taken twice by the same researcher, and the average was recorded. Lymphedema was defined as a ≥2-cm circumferential difference at any measurement point compared to the contralateral limb, consistent with hospital protocol. 2) Upper limb function: assessed using the Disabilities of the Arm, Shoulder, and Hand (DASH) questionnaire. The scale comprises 30 items (21 on disability and nine on symptoms), each scored 0–4 (0 = no difficulty, 4 = unable). Total score = [(Sum of responses/number of answered items) − 1] × 25 (Standard scoring formula: Confirm if this was used or provide the formula used). Range: 0–100; higher scores indicate greater disability. 3) Quality of life (QoL): assessed using the Functional Assessment of Cancer Therapy-Breast (FACT-B) scale (Version 4). The 36-item scale covers Physical Well-Being (PWB; seven items), Social/Family Well-Being (SWB; seven items), Emotional Well-Being (EWB; six items), Functional Well-Being (FWB; seven items), and Breast Cancer Subscale (BCS; nine items). Items were scored 0–4 (0 = not at all, 4 = very much). Total score = PWB + SWB + EWB + FWB + BCS. Range: 0–144; higher scores indicate better QoL. Validated Chinese versions of the DASH and FACT-B scales were used. 4) Exercise compliance: Patients recorded exercise type, duration, frequency, and subjective feelings daily. Compliance was self-reported and verified by researchers via weekly WeChat follow-up. Compliance (%) = (Actual completed exercise/Planned exercise) × 100%. Adequate compliance was defined as achieving ≥80% of the planned weekly exercise volume.

### 2.4 Sample size estimation

We performed *a priori* sample size calculation using the PASS 2020 software. Based on preliminary data from our institution, we assumed a lymphedema incidence of 35% in the control group. To detect a 20% absolute reduction (to 15%) in the experimental group with 80% power and a two-sided alpha of 0.05, a minimum of 48 participants per group was required. Accounting for an anticipated 10% dropout rate, we aimed to recruit 52 participants per group, yielding a total sample size of 104.

### 2.5 Statistical analysis

Statistical analyses were performed using SPSS 26.0. Continuous data are presented as mean ± standard deviation (SD) and were compared using independent samples t-tests, following the confirmation of normality with the Shapiro–Wilk test and homogeneity of variances with Levene’s test. Categorical data are presented as frequencies (percentages) and were compared using chi-square (χ^2^) tests. In addition to p-values, effect sizes were calculated to enhance clinical interpretability: mean differences (MDs) with 95% confidence intervals (95% CIs) for continuous outcomes (DASH and FACT-B scores), and absolute risk reductions (ARRs) with 95% CI for categorical outcomes (lymphedema incidence). A p-value <0.05 was considered statistically significant.

## 3 Results

### 3.1 Incidence of lymphedema

The incidence of lymphedema was significantly lower in the experimental group (7.69%, 4/52) compared to the control group (34.62%, 18/52), with an absolute risk reduction of 26.92% (95% CI: 12.51% to 41.33%). See **Table 2**.

**Table 2:** Incidence of upper limb lymphedema, upper limb function, and quality of life scores in the two groups.

Group	n	Lymphedema incidence n (%)	DASH score (mean ± SD)	FACT-B score (mean ± SD)	Between-group difference (95% CI)
Control group	52	18 (34.62)	31.2 ± 7.1	70.2 ± 6.3	
Experimental group	52	4 (7.69)	18.3 ± 5.3	83.5 ± 6.7	
Effect estimate					
- Lymphedema					−26.92% (−41.33% to −12.51%) ^*^
- DASH					−12.90 (−15.80 to −10.00)
- FACT-B					+13.30 (10.49 to 16.11)

DASH, Disabilities of the Arm, Shoulder, and Hand; FACT-B, Functional Assessment of Cancer Therapy-Breast.

^*^For lymphedema incidence, the between-group difference represents the absolute risk reduction (ARR) and its 95% CI, calculated using the Newcombe–Wilson method without continuity correction.

### 3.2 Upper limb function and quality of life

The DASH score was significantly lower in the experimental group (18.3 ± 5.3) than in the control group (31.2 ± 7.1), with a mean difference of −12.90 points (95% CI: −15.80 to −10.00). The FACT-B score was significantly higher in the experimental group (83.5 ± 6.7) than in the control group (70.2 ± 6.3), with a mean difference of +13.30 points (95% CI: 10.49 to 16.11). See **Table 2**.

### 3.3 Exercise compliance

The weekly exercise compliance rate was significantly higher in the experimental group (96.15%, 50/52) compared to the control group (78.85%, 41/52) (χ^2^ = 3.911, p = 0.027). See **Table 3**.

**Table 3:** Home exercise compliance rates.

Group	n	Complete compliance n (%)	Partial compliance n (%)	Non-compliance n (%)	Compliance rate n (%)
Control group	52	35	6	11	41 (78.85%)
Experimental group	52	41	9	2	50 (96.15%)
Statistic (χ^2^)					3.911
p-Value					0.027

Overall compliance rate includes complete + partial compliance as defined by achieving ≥80% of planned exercise.

## 4 Discussion

This pre–post controlled study demonstrated that a structured, consensus-based home exercise intervention significantly reduced the short-term incidence of BCRL, improved upper limb function, and enhanced quality of life in postoperative patients. Notably, our intervention was explicitly structured according to the recently published “Expert Consensus on Home Exercise for Prevention and Treatment of Lymphedema in Postoperative Breast Cancer Patients” (4), which enhances the clinical relevance and standardization of our approach.

The observed absolute risk reduction of 26.92% (95% CI: 12.51% to 41.33%) in lymphedema incidence underscores a substantial clinical benefit. This aligns with the findings of Cheng L. et al. (7), whose meta-analysis suggested that structured exercise can reduce lymphedema risk by 40%–60%. The underlying mechanism likely involves the muscle pump effect, enhancing lymphatic return: resistance training directly compresses lymphatic vessels during muscle contraction, while aerobic exercise improves systemic circulation, indirectly optimizing lymphatic flow efficiency (8). Furthermore, the deep breathing exercises and self-MLD incorporated in this intervention likely augmented lymphatic return by modulating intrathoracic pressure and providing direct physical stimulation to lymphatic pathways (9).

Notably, our intervention was explicitly structured based on the Consensus. Key elements, such as controlling resistance exercise intensity at 50%–80% 1RM and frequency at 2–3 times/week, may be crucial for its superior efficacy compared to non-systematic approaches. This structured program achieved a lymphedema incidence rate of 7.69% (non-incidence rate 92.31%), surpassing the preventive efficiency reported in less structured interventions, such as the study by Schmitz K.H. et al. (10), further validating the clinical value of standardized exercise protocols.

The experimental group exhibited a 41.3% reduction in DASH score (indicating improved function) and a 19.0% increase in FACT-B score (indicating enhanced QoL) compared to controls. Moreover, the mean differences in DASH (−12.90 points) and FACT-B (+13.30 points) scores, both with 95% confidence intervals excluding zero, strongly support not only statistical significance but also a clinically meaningful improvement from the patient’s perspective. From a functional perspective, resistance training strengthens rotator cuff and upper limb muscles, mitigating post-surgical joint adhesions, while flexibility training (e.g., yoga and stretching) improves soft tissue extensibility, alleviating movement restrictions (11). The inclusion of such flexibility and mind–body practices is strongly supported by contemporary research; an integrative review confirmed that yoga interventions specifically demonstrate beneficial effects on lymphedema symptoms, upper limb mobility, and psychosocial well-being in breast cancer survivors (12). This external validation reinforces the rationale for including these components in structured exercise protocols to comprehensively address both physical and psychological sequelae of BCRL. These findings corroborate those of Michels D. et al. (13), who documented 30%–40% improvements in upper limb function scores with structured exercise.

The QoL improvement likely stems from multi-faceted effects: aerobic exercise alleviates depressive mood via endorphin release, resistance training enhances self-efficacy, and multidisciplinary support reduces disease-related uncertainty (14). Notably, the largest improvement in the FACT-B was observed in the Social/Family Well-Being dimension (~22%), suggesting that improved physical function enables patients to engage more actively in social roles. This aligns with the “function-psychology-society” positive cycle model proposed by Klein I. et al. (15).

Exercise compliance was significantly higher in the experimental group (96.15% *vs*. 78.85%), underscoring the importance of multidisciplinary collaboration and structured support. Within the team, exercise therapists tailored plans, lymphedema therapists ensured safe self-MLD technique, and oncology nurses reinforced adherence via WeChat follow-up. This interprofessional approach aligns with the “interprofessional management” model advocated by the American Academy of Oncology Physical Rehabilitation (AOSR) (16) and is consistently associated with 20%–30% higher compliance compared to single-discipline management (17). Our findings are further supported by recent evidence on health coaching, which has been shown to improve adherence and self-management among cancer survivors with lymphedema by providing sustained motivation and personalized guidance (18). The weekly WeChat follow-up in our study functionally acted as a form of digital health coaching, creating a “behavioral feedback loop” that likely contributed to the exceptional compliance rates observed.

The use of structured tools (manuals and videos) mitigated limitations posed by healthcare resource constraints. Standardized diagrams and video demonstrations simplified execution for patients, while the WeChat “behavioral feedback loop” (weekly checks) facilitated closed-loop management. This resonates with the WHO’s “digital health intervention” principle, leveraging technology to improve chronic disease management accessibility (19).

It is important to consider, however, that the experimental group received not only the structured exercise but also enhanced multidisciplinary support and digital follow-up via WeChat. This additional attention and support, distinct from the exercise itself, may have contributed to the improved outcomes, representing a potential confounding factor. Future studies should aim to disentangle the specific effects of the exercise program from those of the supportive care framework.

This study has several limitations. First, although baseline characteristics were comparable, the non-randomized chronological assignment may introduce selection bias or temporal confounding. Future studies should employ randomized designs to strengthen causal inference. Second, participants were recruited from a single oncology center, which may limit generalizability to primary care settings or diverse populations. Future studies should include institutions across different healthcare levels. Third, the short follow-up period (1–2 months) limits our ability to assess the long-term incidence of lymphedema; future studies should include follow-up at 12 and 24 months to evaluate sustained effects. Fourth, the study did not evaluate the individual contributions of different exercise components (e.g., resistance *vs*. aerobic). Future trials should incorporate factorial or component-analysis designs to identify the most effective elements of the exercise program. Finally, the specific impact of compression sleeves in high-risk patients remains unclear, warranting further investigation into risk-stratified interventions. Additionally, exercise compliance was self-reported and verified via WeChat, which may introduce reporting bias.

## 5 Conclusion

The consensus-based systematic home exercise intervention effectively reduced lymphedema incidence, improved upper limb function, and enhanced quality of life in breast cancer patients, demonstrating high clinical feasibility. We recommend the clinical application and promotion of this Consensus-based program, supported by multidisciplinary teams and structured supervision. Future multi-center randomized controlled trials are needed to confirm the long-term efficacy of this approach.

## Data Availability

The original contributions presented in the study are included in the article/**Supplementary Material**. Further inquiries can be directed to the corresponding author.
