# Supplementary material for: Effect of home exercise on prevention and treatment of lymphedema in breast cancer patients
Source: Front Oncol. 2025 Dec 16;15:1665012. doi: 10.3389/fonc.2025.1665012 (PMC12751289; doi:10.3389/fonc.2025.1665012)
Supplement: Supplementary file 1 [file Table1.docx]

**Structured Home Exercise Protocol for Prevention and Treatment of Breast Cancer-Related Lymphedema**

| **Component** | **Key Exercises / Modalities** | **Intensity & Parameters** | **Frequency & Duration** | **Progression & Safety Instructions** |
| --- | --- | --- | --- | --- |
| **Resistance Training** *Aim: Improve muscle strength and pump function* | • Bodyweight exercises (e.g., squats).  • Exercises with equipment: resistance bands, dumbbells. | • Intensity: 50-80% of 1-Repetition Maximum (1RM).  • Volume: 8-12 repetitions per exercise, 2-3 sets.  • Rest: 60 seconds between sets. | • Frequency: 2-3 non-consecutive days per week. | • Progression: Increase resistance when 12 repetitions can be completed with good form for 2 consecutive sessions.  • Safety: Exhale during exertion (muscle contraction), inhale during relaxation. Patients with confirmed lymphedema should avoid high-resistance, repetitive sports (e.g., rowing, tennis). |
| **Flexibility Training**  Aim: Improve range of motion and soft tissue extensibility | • Yoga, Pilates, Qigong, stretching exercises.  • Often integrated with breathing techniques. | • Intensity: Stretch to the point of mild tension, not pain.  • Hold Time: 20-30 seconds per stretch.  • Repetitions: 2-3 times per stretch. | • Frequency: 2-3 days per week.  • Session Duration: 30-60 minutes. | • Progression: Gradually increase the range of motion as tolerance improves.  • Safety: Avoid bouncing. Movements should be slow and controlled. Adjust difficulty based on patient acceptance and ability. |
| **Aerobic Exercise**  Aim: Enhance systemic circulation and cardiopulmonary fitness | • Brisk walking, jogging, cycling, hiking, Tai Chi, stair climbing. | • **Intensity:** Moderate (able to talk but not sing). Can be calculated using the Heart Rate Reserve (HRR) method: Target Heart Rate = [(220 - age) - resting HR] × (40%-60%) + resting HR. • **Borg Scale:** 12-15 ("somewhat hard" to "hard"). | • **Frequency:** 3-5 days per week. • **Duration:** 30-60 minutes per session or 150-300 minutes weekly. | • Progression: First increase duration, then intensity.  • Safety: High-risk patients are recommended to wear a properly fitted compression sleeve during exercise. |
| **Deep Breathing Exercises**  Aim: Modulate intra-thoracic pressure to facilitate lymphatic return | • Diaphragmatic breathing. | • Technique: Deep inhalation during muscle contraction, exhalation during muscle relaxation.  • Repetitions: 5-10 deep breaths per set. | • **Frequency:** Daily, integrated into other exercises or performed as a standalone practice. | • **Safety:** Ensure a relaxed posture. Do not force the breath. |
| **Self-Manual Lymphatic Drainage (Self-MLD)**  Aim: Stimulate lymphatic pathways and redirect fluid | • Light, circular massage movements in defined sequences (e.g., starting at the neck, moving to the upper arm). | • Pressure: Very light, skin-stretching pressure only.  • Duration: 15-20 minutes per session. | • **Frequency:** Twice daily (morning and evening) OR once post-exercise. | • **Safety:** Must be taught by a certified lymphedema therapist. Perform on dry, unbroken skin. Elevate the affected limb if possible. Avoid areas with active infection or thrombosis. |

**Note:** This protocol is based on the *Expert Consensus on Home Exercise for Prevention and Treatment of Lymphedema in Postoperative Breast Cancer Patients* . All exercises should be initiated only after clearance from the healthcare team and under guidance. Patients were advised to perform exercises in a pain-free range and to stop immediately if they experienced any sharp pain, dizziness, or excessive shortness of breath.
